# Silencing collapsin response mediator protein-2 reprograms macrophage phenotype and improves infarct healing in experimental myocardial infarction model

**DOI:** 10.1186/s12950-015-0053-8

**Published:** 2015-02-10

**Authors:** Long-Shu Zhou, Guo-Long Zhao, Qiang Liu, Shu-Cai Jiang, Yun Wang, Dong-Mei Zhang

**Affiliations:** Department of Cardiovascular Surgery, General Hospital of Ningxia Medical University, Ningxia Medical University, Yinchuan, 750004 People’s Republic of China; Department of Neurosurgery, General Hospital of Ningxia Medical University, Ningxia Medical University, Yinchuan, 750004 People’s Republic of China; Department of Anesthesiology, General Hospital of Ningxia Medical University, Ningxia Medical University, Yinchuan, 750004 People’s Republic of China

**Keywords:** Collapsin response mediator protein-2, Macrophages, Phenotypes, Inflammation, ApoE^−/−^, Myocardial infarction, Fibrosis

## Abstract

**Background:**

Delayed M1 toward M2 macrophage phenotype transition is considered one of the major causes for the impaired healing after myocardial infarction (MI). While searching for molecules that modulate M1 and M2 macrophage polarization, we identified collapsin response mediator protein-2 (CRMP2) as a novel molecule involved in macrophage polarization to M1. In this study, we evaluated the effect of silencing CRMP2 on macrophage polarization, inflammation and fibrosis post myocardial infarction.

**Methods:**

CRMP2 expression was assessed with Western blotting or immunohistochemistry. Macrophage phenotypes were measured with flow cytometry, quantitative real-time PCR (qPCR), Western blotting or immunohistochemistry. CRMP2 siRNA was delivered into the macrophages infiltrated in the wound of ApoE^−/−^ mice through lipidoid nanoparticle, and fibrosis, leukocyte infiltration and inflammation parameters were measured with qPCR. Infarct size was measured with Masson’s trichrome staining. Echocardiography was performed to assess ventricular systolic dimension, left ventricular diastolic dimension, anterior wall thickness and posterior wall thickness. Student’s t-test (for 2 groups) and ANOVA (for > 2 groups) were used for statistical analyses.

**Results:**

CRMP2 was expressed in a higher level in M1 macrophages than M2 subsets, and CRMP2 RNA interference (RNAi) resulted in a switch of bone marrow-derived macrophages from M1 to M2 phenotype. High level of CRMP2 was also observed in the macrophages infiltrated in the infarct area 3 days post MI in both wildtype (WT) and ApoE^−/−^ mice, and the expression of CRMP2 retained in the infiltrated macrophages of ApoE^−/−^ mice but not in that of WT mice 10 days after MI. Nanoparticle-mediated delivery of CRMP2 siRNA to ApoE^−/−^ mice with MI resulted in dramatic switch of wound macrophages from M1 to M2 phenotype, marked decrease in inflammation and fibrosis, and significant attenuation of post-MI heart failure and mortality.

**Conclusion:**

CRMP2 is highly expressed in M1 macrophages and silencing CRMP2 reprograms macrophage phenotype and improves infarct healing in atherosclerotic mice.

## Introduction

Myocardial infarction (MI) and the resulting complications are a major cause of death worldwide. Following MI, circulating blood monocytes respond to chemotactic factors, migrate into the infarct myocardium, and differentiate into macrophages. At the injury site, macrophages remove necrotic cardiac myocytes and apoptotic neutrophils; secrete cytokines, chemokines, and growth factors; and modulate phases of the angiogenic response. As such, macrophage is a primary responder cell type that is involved in the regulation of post-MI wound healing at multiple levels. Two phases of infiltration are defined after MI. In the first few days after injury, inflammatory monocytes and classical M1 macrophages rapidly invade the wound to defend against pathogens, phagocytose, and lyse debris, and thus pave the way for tissue regeneration [1]. During subsequent healing, classical macrophages retreat and give way to M2 macrophages, which exhibit a less inflammatory panel of functions that supports tissue repair and regeneration [2-4]. Recent studies have indicated that anti-inflammatory strategies targeting inflammatory monocyte/macrophage subsets could reduce excessive inflammation and improve cardiovascular outcomes [5-8]. Thus, identification of targets that modulate macrophage phenotypes and functions may lead to development of novel therapeutic approaches for MI.

In an effort of searching for molecules involved in macrophage polarization, we established a phenotypic screening assay and screened molecules that are able to switch M1 to M2 using pooled shRNA library. Collapsin response mediator protein-2 (CRMP2), a multifunctional adaptor protein first described in the CNS and thoroughly studied in neurons [9-11], was identified as a novel protein potentially involved in macrophage polarization. CRMP2 belongs to a family of five homologous members and was first identified as a mediator of semaphorin-induced growth-cone collapse [10]. Downstream of semaphorin signal, they reorganize the cytoskeleton by controlling microtubule assembly [12-14], thereby playing a crucial role in axonal outgrowth and neurite extension [10]. In recent years, several observations have shown that CRMP2 is present in the immune system and plays a critical role in T lymphocyte polarization and migration [15-17]. CRMP2 has the ability to bind to the cytoskeletal elements tubulin and vimentin redistributed at the uropod, the flexible structure of T cells, and RNA mediated crmp2/dpysl2 gene silencing and blocking antibody strongly reduces T cell polarization (uropod formation) and migration. Conversely, increased CRMP2 expression promotes uropod formation and the migration of transfected lymphocytes [16]. Thus, CRMP2 is a key player in cell behavior within a wider field than just the nervous system.

In the present study, we observed that CRMP2 was expressed in a significantly higher level in M1 macrophages than M2 macrophage subsets, and knockdown of CRMP2 with RNA interference (RNAi) in M1 macrophages resulted in a significant decrease in M1 gene expression and increase in M2 gene expression. High level of CRMP2 was also observed in the macrophages infiltrated in the infarct area 3 days post MI in both wildtype (WT) and ApoE^−/−^ mice, and the expression of CRMP2 retained in the infiltrated macrophages of ApoE^−/−^ mice but not in that of WT mice 10 days after MI. In ApoE^−/−^ mice with MI, nanoparticle-mediated delivery of CRMP2 siRNA to wound macrophages efficiently suppressed expression of CRMP2 *in vivo*, associated with reduced expression of inflammatory M1 macrophage markers, increased resolution of inflammation, accelerated infarct healing, and attenuated fibrosis, development of post-MI heart failure and mortality.

## Materials and methods

### Animal models

C57BL/6 J and B6.129P2-Apoetm1Unc/J (ApoE^−/−^) mice used in this study were purchased from The Jackson Laboratory (Bar Harbor, ME, USA). ApoE^−/−^ mice were fed on a high-cholesterol diet for 6 months (Harlan Teklad, 0.2% total cholesterol). Myocardial infarction was induced by permanent coronary ligation [18]. Briefly, animals were anesthetized using a mixture of ketamine, xylazine and atropine (100, 20 and 1.2 mg/kg, respectively, i.p.) and mechanically ventilated. Under a surgical microscope, left thoracotomy was performed to expose the heart. The left coronary artery was identified and ligated with a 7–0 silk suture at a level approximately 2 mm below the edge of the left auricle. Sham operation was also performed without ligating the coronary artery. After surgery mice were monitored daily for 4 weeks and autopsy was performed on all mice found dead to identify the cause of death such as post-MI cardiac rupture or heart failure, as described previously [18]. Some mice were sacrificed at 6 and 24 h, 3, 7 and 14 days, and 4 weeks, respectively, following MI. Infarct and non-infarct myocardium were separated and snap frozen in liquid nitrogen and stored at −80°C for molecular assays. Further, some hearts were fixed in 10% formalin or fresh frozen for embedding with OCT for histological analyses. All animal studies were approved by the Institutional Animal Care and Use Committee (IACUC) of Ningxia Medical University.

### Culture of bone marrow-derived macrophages

Bone marrows were obtained from 8–12 week old C57BL/6 mice, and bone marrow-derived macrophages (BMDMs) were cultured using a previously described protocol [19]. In brief, bone marrow cells were flushed out from mouse tibias and femurs with Dulbecco’s modification of Eagle’s medium (DMEM; Gibco BRL, Shanghai, China) containing 100 μg/ml primocin (Invivogen, San Diego, CA, USA) and then filtered through 100-μm cell strainer (BD, Shanghai, China). Red blood cells were removed with lysis buffer (0.75% NH4Cl, 0.02% Tris–HCl, pH 7.2) on ice for 15 min. Bone marrow cells were cultured in a DMEM supplement with 15% fetal calf serum (Sigma-Aldrich, Shanghai, China) and 15% L929 conditioned medium and seeded in 75-mm2 flasks at 37°C in 5% CO2 incubator for 5 days. L929 is a murine fibroblast cell line that produces M-CSF and has been used widely for macrophage differentiation studies [20].

### Activation of BMDMs

BMDMs were seeded in six-well plates at a density of 1 × 10^6^ cells/well for overnight. Cells were then activated as follows: M1, IFN-γ (100 ng/ml; R&D Systems, Shanghai, China) and LPS (50 ng/ml, Sigma-Aldrich); M2a, IL-4 (20 ng/ml; R&D Systems, Shanghai, China); M2b, immune complex (150 μg/ml anti-chicken egg albumin (ovalbumin) monoclonal antibody preincubated with 15 μg/ml ovalbumin at 37°C for 30 min, Sigma-Aldrich) and LPS (50 ng/ml); and M2c, IL-10 (20 ng/ml, R&D Systems). Seven and 24 h later, cells were harvested for total RNA or protein extraction. Trypan blue staining did not reveal significant cell death in activated BMDMs at both time points. To collect the supernatants, cells were activated with appropriate stimuli for 7 h. The cells were then washed with phosphate-buffered saline (PBS) and incubated with serum-free DMEM. Forty-eight hours later, supernatants were collected and stored in −80°C until use.

### Construct and RNA interference

Three CRMP-2 siRNAs (siCRMP-2-1, siCRMP-2-2 and siCRMP-2-3) were designed to target mouse CRMP-2 mRNA (NM_009955) sequences: 5’-ACUCCUUCCUCGUGUACA-3′, 5’-GAUGGGUUGAUCAAGCAA-3′ and 5’-ACTCCTTCCTCGTGTACAT-3’ respectively. A non-targeting siRNA was used as a negative control for all siRNA transfection experiments. All siRNAs were synthesized by Shanghai GenePharma (Shanghai, China). The efficacy and specificity of siRNAs were determined as described previously [21].

### Lipidoid-based siRNA formulations and intravenous injection

Nanoparticles were prepared with the cationic lipid C12-200, disteroylphosphatidyl choline, cholesterol, and PEG–DMG using a spontaneous vesicle formation formulation procedure [22]. In brief, lipids were dissolved in 90% ethanol solution and mixed with siRNA solution (25 mM citrate, pH 3 ratio) at fixed speed (1:1 ratio) and diluted immediately with PBS to final 25% ethanol. The ethanol was then removed and the external buffer replaced with PBS (155 mM NaCl, 3 mM Na2HPO4, 1 mM KH2PO4, pH 7.5) by dialysis. The final lipid:siRNA weight ratio was ∼ 7:1. Particle size and zeta potential were determined using a Malvern Zetasizer NanoZS (Malvern, UK). siRNA content was determined by ion exchange HPLC (Agilent) assay using DNAPac Pa200 column (Dionex Corporation Dionex, 260 nm, 55°C run at 2 mL/min). siRNA entrapment efficiency was determined by the Quant-iT RiboGreen RNA assay (Invitrogen, Carlsbad, CA) according to the manufacturer’s instruction. For intravenous injection, mice were anesthetized and injected through the tail vein with 0.5 mg/kg of either siCRMP2 or control siRNA (siCON) incorporated into lipidoid nanoparticles (LNPs).

### Plasmids and adenoviral infection

IRF expression construct was generated in the pENTR vector (Invitrogen) modified to contain the CMV promoter and IRES-linked GFP (pBent) as previously described [23]. For delivery into mouse bone marrow-derived macrophages, IRF5/luciferase cassettes were excised and subcloned into the pBent vector, modified to contain CMV-driven GFP in the orientation opposite to the luciferase gene and recombined into pAD/PL DEST vector (Invitrogen) for adenovirus production. Adenoviral infections of mouse bone-marrow-derived macrophages were performed in 96-well plates in triplicate. The plates with serum-free RPMI medium 1640 containing the desired number of viral particles were centrifuged at 400 g for 30 min then placed at 37°C overnight. The next day the virus media were replaced with 100 μL of standard media and the cells were allowed to recover for 2 days before the application of experimental conditions.

### Quantitative RT-PCR

Total messenger RNA (mRNA) was extracted using the RNeasy Micro Kit (Qiagen) according to manufacturer’s instructions. One microgram of mRNA was reverse transcribed using the high capacity RNA to cDNA kit (Applied Biosystems). TaqMan gene expression assays (Applied Biosystems) were used to quantify target genes. The relative changes were normalized to Gapdh mRNA using the 2-∆∆CT method.

### Western blot analysis

Myeloid cells from heart tissue were isolated as described above, washed with ice-cold PBS and homogenized on ice using RIPA lysis buffer (Millipore) supplemented with complete protease inhibitor cocktail (Roche). Protein concentration was measured using BCA assay (Pierce). Samples of 15 μg were loaded on 10% SDS-PAGE and transferred onto PVDF membranes (Bio-Rad). Membranes were blocked with 5% non fat dry milk in TBS-Tween 0.1% and incubated with primary antibodies against the specific protein and peroxidase-coupled secondary antibodies. β-actin was used as control. Signals were visualized with enhanced chemiluminescence detection system (ECL Plus, Amersham Life Science), and densitometric analysis was performed with ImageJ 1.40 g (National Institutes of Health). The following antibodies were used: rabbit polyclonal anti-CRMP2 (Abcam), rabbit polyclonal anti-IRF5 (Abcam), mouse monoclonal anti-IRF4 (eBioscience), rabbit polyclonal anti-IRF3 (Abcam), rabbit monoclonal anti-CD86 (Abcam), rabbit polyclonal anti-CD163 (Santa Cruz Biotechnology).

### Histology

To eliminate blood contamination, hearts were perfused with ice-cold PBS after mice were euthanized. Hearts were removed, rinsed in PBS, embedded in O.C.T. compound (Sakura Finetek), and frozen in an isopentane bath on dry ice. For immunofluorescence staining, sections (5 μm) were stained with mixture of antibodies, including mouse monoclonal anti-CD11b antibody (clone M1/70, BD Biosciences) and a rabbit polycolonal anti-iNOS (Abcam), mouse monoclonal anti-CD11b antibody (BD Biosciences) and a rabbit polycolonal anti-IGF-1 (Abcam), or mouse monoclonal anti-CD11b antibody (BD Biosciences) and a rabbit polycolonal anti-CRMP2 (Abcam), followed by incubation with a mixture of Alexa Fluor® 488 Goat anti-mouse IgG and Cy3-conjugated anti-rabbit IgG. The slides were cover slipped using a mounting medium with DAPI (Vector Laboratories, Inc.) to identify nuclei. Images were observed and captured using Nikon Eclipse 80i with a Cascade Model 512 B camera (Roper Scientific). For immunohistochemistry, histology of the heart was performed on day 7 after MI in ApoE^−/−^ mice. Frozen sections (5 μm) were stained for antibodies against CD11b (BD Biosciences), CD86 (Abcam), and CD206 (Abcam). The appropriate biotinylated secondary antibodies, ABC kit (Vector Laboratories, Inc.) and AEC substrate (Dako) were used for color development, and all the sections were counterstained with Harris hematoxylin. The slides were scanned by a digital slide scanner, NanoZoomer 2.0-RS in 40x high resolution mode (Hamamatsu, Japan). The positive area was quantified using IPLab (version 3.9.3; Scanalytics, Inc.) and analyzing five high power fields per section and per animal.

### Postmortem histological determination of scar area

Mice were euthanized by cervical dislocation after anesthesia with 5% isoflurane for histological assay at 4 weeks after MI [24]. Hearts were embedded in paraffin after being fixed in 4% paraformaldehyde. Serial sections (5 mm thickness) were performed Masson’s trichrome stain to detect scar area and fibrosis in cardiac muscle. Computerized morphometry was used to calculate the scar extent as the ratio of scar and total left ventricular area using Imaging Pro Plus software.

### Echocardiographic studies of cardiac function

Echocardiography was performed to assess the cardiac function after MI in a blinded manner [25]. At 2 days post operation (POD) and weekly until sacrificed, Mice were anesthetized (2% isoflurane and oxygen) and put in a supine position. Both two-dimensional and M-mode images were recorded using a 30-MHz transducer. Left ventricular systolic dimension (LVDs), left ventricular diastolic dimension (LVDd), anterior wall thickness (AWT) and posterior wall thickness (PWT) were measured to calculate left ventricular ejection fraction (LVEF) and fractional shortening (FS) as an average of three beats.

### Statistics

Data are expressed as mean ± sem. Analyses were performed using Prism 6.0a (GraphPad Software Inc.). The group means were compared using a Student’s t-test (for 2 groups) and ANOVA, followed by Bonferroni post-tests (for > 2 groups). P values of <0.05 indicate statistical significance.

## Results

### M1 macrophages expressed higher level of CRMP2 than M2 subsets

**Figure 1:**
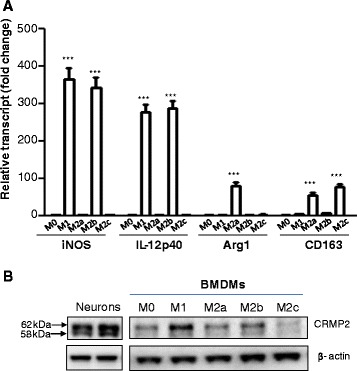
**Expression of CRMP2 in BMDM subsets. A**. BMDMs were incubated with IFN-γ + LPS (M1), IL-4 (M2a), IC+ LPS (M2b), or IL-10 (M2c) for 7 h. The mRNA levels of iNOS, IL12p40, Arg-1, and CD163 genes were detected by qPCR. Data mean ± SEM of gene fold changes against untreated M0 cells. ***P < 0.001 vs. untreated M0 cells. **B**. Western blot analysis of CRMP2 with a specific antibody showed higher level of CRMP2 expression in M1 versus M2 and M0 macrophage subsets. The immunoblots of β-actin was used as a loading control. Shown are representative data from three independent experiments with similar results.

Before examine the expression of CRMP2 in different subsets of macrophages, we first confirmed the phenotypes of polarized BMDMs by measuring their distinct gene expression profile using real-time RT-PCR (Figure 1A). IFN-γ and LPS-induced M1 macrophages expressed high levels of iNOS and IL-12p40 but low level of IL-10 and arginase-1 (Arg1) (Figure 1B). M2a macrophages (induced by IL-4) expressed high levels of Arg1 but low levels of iNOS and IL-12p40 (Figure 1A). M2b macrophages (treated with immune complex and LPS) also expressed iNOS and IL-12p40 but at lower levels as compared to M1 macrophages (Figure 1A). M2c macrophages (induced by IL-10) expressed CD163 but not iNOS, and very low IL-12p40 (Figure 1A). These results, consistent with the previous reports [26], indicated successful polarization of BMDMs to M1 and M2 subsets. We next examined the expression of CRMP2 in different subsets of macrophages using Western blot analysis. As shown in Figure 1B, CRMP2, exhibiting two major products with apparent molecular mass of 58 and 62 kDa, was detected in all subsets of macrophages but with remarkably higher level in M1 than in M2 subtypes. Using primary neuronal cultures for Western blotting, we also detected two bands as shown in the immunoblots of CRMP2 in macrophages, thereby confirming the expression of CRMP2 in macrophages. While the 62 kDa product corresponds to the native protein (theoretical molecular weight of 62.5 kDa), the 58 kDa band is likely the proteolytic cleavage. We also examined the expression of CRMP1 and CRMP3-5 in macrophages and found that very low level of these CRMPs was detected and their expression was not changed during the differentiation and polarization of macrophages (data not shown). Thus, these data suggest that CRMP2 is specifically expressed in M1 macrophages.

### Knockdown of CRMP2 converted BMDMs from M1 to M2 macrophages

**Figure 2:**
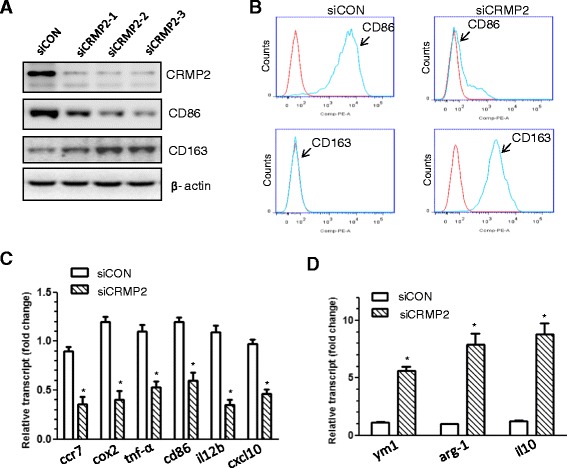
**CRMP2 RNAi promoted M1 to M2 switch. A**. Western blot analysis indicated that knockdown of CRMP2 with three siRNAs in M1 macrophages resulted in marked decrease in the expression of CD86 but increase in the expression of CD163. Shown are representative data from three independent experiments with similar results. **B**. Flow cytometry images showed that CRMP2 RNAi with a mixture of three siRNAs in M1 macrophages resulted in a marked decrease in the expression of CD86 but an increase in the expression of CD163. Shown are representative data from three independent experiments with similar results. **C** & **D**. Gene expression analysis of selected M1- and M2-associated transcripts in macrophages after being treated with control siRNA or CRMP2 siRNA. Data were mean ± SEM from three independent experiments. *p < 0.05 compared with control.

Given the higher level of CRMP2 expression in M1 than M2 macrophages, we attempted to examine whether CRMP2 RNAi in M1 macrophages affects their phenotype. M1 BMDMs were transfected with three different CRMP2 siRNAs and levels of CRMP2 were assessed with Western blotting. As shown in Figure 2A, M1 macrophages transfected with each of the three siRNAs (siCRMP2-1, −2, and −3) exhibited markedly reduced expression of CRMP2 compared the cells transfected with the control siRNA (siCON). Therefore, in the following studies, a combination of the three siRNAs, exhibited as siCRMP2, was used. Using flow cytometry, we observed that CRMP2 RNAi resulted in a marked decrease in the expression of CD86, a typical M1 macrophage marker, and a marked increase of CD163, a typical marker of M2 macrophages [26] (Figure 2B). Moreover, by measuring the expression profile of M1 and M2 macrophage genes, we observed that CRMP2 RNAi resulted in decreased expression of a panel of M1 genes, including ccr7, cox2, tnf-α, cd86, il12b, and cxcl10, and in the increase of a number of M2 genes, including ym1, arg-1, and il-10 (Figure 2C and D). Though more studies are needed to further define the M2 subtype (M2a or M2c) that was converted from M1 by the CRMP2 knockdown, based on the increase in arg-1 expression, it is postulated that CRMP2 knockdown may convert M1 to M2a phenotype [27].

### Downregulation of IRF5 was responsible for the CRMP2 RNAi-induced switch of M1 to M2 macrophages

To understand the mechanism underlying the modulation of macrophage phenotype by CRMP2, we first examined whether CRMP2 RNAi affects the expression of IRF5, a transcription factor known to play a key role in promoting macrophage polarization to M1 [23]. Western blotting of IRF5 exhibited a major product with the molecular mass of 56 kDa, and CRMP2 knockdown resulted in a marked decrease in the level of IRF5. However, the expression of IRF4, which controls M2 polarization in mice [28], was not affected by CRMP2 knockdown in the M1 macrophages. The expression of IRF3, another member of the IRF family central to the innate immune response, was not affected by CRMP2 knockdown as well. These data suggest that CRMP2 RNAi resulted in a specific down-regulation of IRF5 in M1 macrophages.

**Figure 3:**
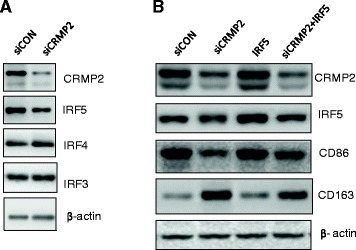
**IRF5 was involved in CRMP2-mediated polarization of macrophages. A**. Representative immunoblots showed that CRMP2 RNAi in M1 macrophages resulted in a decrease in IRF5 expression, without affecting the expression of IRF3 and IRF4. **B**. Representative immunoblots showed that CRMP2 RNAi in M1 macrophages resulted in decreased expression of CD86 but increased expression of CD163, which were reversed by the overexpression of IRF5. Shown are representative data from three independent experiments with similar results.

To explore the possible involvement of IRF5 in the regulatory role of CRMP2 in macrophage polarization, M1 macrophages were transfected with control or CRMP2 siRNA before being infected with adenoviral constructs containing IRF5 cDNA, and M1/M2 markers were assessed with Western blotting. As shown in Figure 3B, CRMP2 RNAi resulted in decreased expression of CD86 but increased expression of CD163, which was reversed by the IRF5 overexpression.

### CRMP2 was expressed in M1 macrophages infiltrated in the infarct area in both WT and ApoE^−/−^ mice with MI

**Figure 4:**
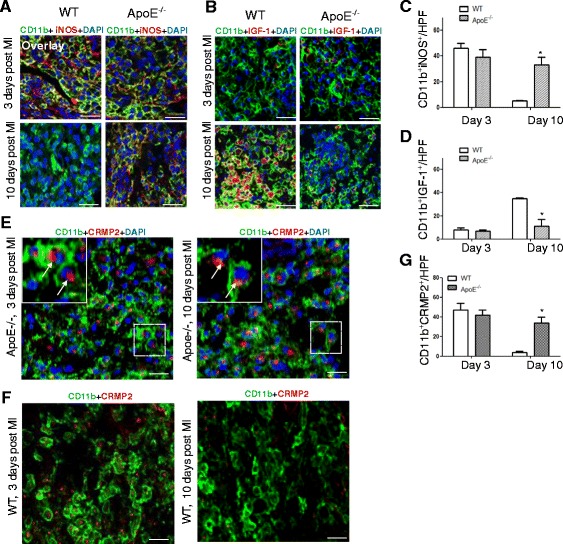
**CRMP2 was predominantly expressed in M1 but not M2 macrophages infiltrated in the wound of wildtype and ApoE** ^**−/−**^ **mice with MI. A** & **B**. Representative confocal microscopy images showed the predominance of M1 (CD11b^+^, iNOS^+^) macrophages 3 days after MI in both wildtype and ApoE^−/−^ mice with MI, and predominance of M2 (CD11b^+^, IGF-1^+^) macrophages in wildtype but predominance of M2 (CD11b^+^, IGF-1^+^) macrophages in ApoE^−/−^ mice 10 days after MI. Scale bar indicates 50 μm. **C**. Quantitation of M1 (CD11b^+^, iNOS^+^) macrophages in the wound 3 and 10 days after MI. **D**. Quantitation of M2 (CD11b^+^, IGF-1^+^) macrophages in the wound 3 and 10 days after MI. **E**. Representative confocal microscopy images showed the expression of CRMP2 in the infiltrated macrophages 3 days after MI in ApoE^−/−^ mice. Arrows indicated localization of CRMP2 in one pole outside of the nucleus. **F**. Representative confocal microscopy images showed the expression of CRMP2 in the infiltrated macrophages 3 days after MI in WT mice. **G**. Quantitation of CRMP2 positive macrophages in the wound 3 and 10 days after MI in WT and ApoE^−/−^ mice. In all of the quantitative experiments, n = 4 mice per group and n = 4 high power fields (hpf). Data are mean ± SEM. *P < 0.05 compared to WT control.

To examine whether CRMP2 is expressed in M1 macrophages *in vivo*, we chose mouse MI model in which M1 macrophages rapidly invade the wound in the first few days after the injury [1]. In addition to wildtype mice, ApoE^−/−^ mice on a high-fat diet were also used to generate MI model. These atherosclerotic mice have impaired resolution of inflammation post-coronary ligation, due to increased and prolonged recruitment of inflammatory monocytes to the heart [29]. The delayed M1 and M2 transition leads to impaired infarct healing [30], mimicking infarct healing in patients with atherosclerosis. M1 and M2 macrophages markers were assessed on 3 and 10 days after MI, respectively. Immunostaining of macrophage markers indicated that the majority of macrophages infiltrated in the wound on day 3 after MI were M1 macrophages (CD11b^+^ and iNOS^+^) in both wildtype and ApoE^−/−^ mice. However, on day 10 after MI, the majority of macrophages infiltrated in the wound were M2 (CD11b^+^ and IGF-1^+^) in wildtype mice but M1 in ApoE^−/−^ mice (Figure 4A-D). These data confirm the notion that M1 macrophages dominate the wound in both the inflammatory and resolution phases post MI. We then examined the expression of CRMP2 in the macrophages infiltrated in the wound. As shown in Figure 4E-G, in ApoE^−/−^ mice with MI, the majority of macrophages infiltrated in the wound expressed CRMP2 on both day 3 and day 10, whereas in WT mice, CRMP2 was expressed in macrophages 3 days after MI, but was modestly expressed in macrophages 10 days after MI. Within the macrophages, CRMP2 was distributed at one pole outside of the nucleus (Figure 4E, arrows indicated). The immunostaining was proved to be specific for CRMP2 and the macrophages markers as no signal was observed when the sections were stained with a control IgG (data not shown). These data suggest that CRMP2 is predominantly expressed in M1 macrophages in the lesion area of MI mice, consistent with its expression pattern in macrophage cultures.

### CRMP2 gene silencing resulted in M1 to M2 switch in macrophages infiltrated in the infarct of ApoE^−/−^ mice with MI

**Figure 5:**
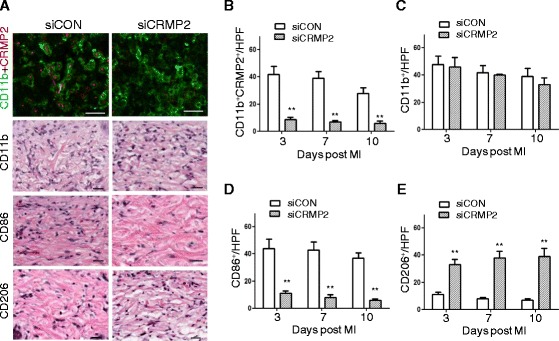
**Silencing CRMP2 promoted macrophage polarization to M2 in the wound of ApoE** ^**−/−**^ **mice with MI. A**. Representative confocal microscopy images showed marked decrease in the expression of CRMP2 in the infiltrated macrophages, in the population of M1 (CD86 positive cells), and in the increase in the population of M2 (CD206 positive cells) in the siCRMP2-treated ApoE^−/−^ mice 3 days after MI. Scale bar indicates 50 μm **B-E**. Quantitation of CRMP2^+^ macrophages (CD11b^+^ and CRMP2^+^), total macrophages (CD11b^+^), M1 macrophages (CD86^+^), and M2 macrophages (CD206^+^) in the wound 3, 7 and 10 days after MI in ApoE^−/−^ mice. n = 4 mice per group and n = 4 high power fields (HPF). Data are mean ± SEM; **p < 0.01 compared to control.

To knockdown of CRMP2 in macrophages *in vivo*, siRNA targeting CRMP2 was delivered to wound macrophages after incorporation into lipidoid nanoparticles [22], which were injected intravenously. Treatment with siCRMP2 in the atherosclerotic mice with MI resulted in efficient knockdown of CRMP2 expression in macrophages on day 3 after MI (Figure 5A and B). Similar changes were also observed on day 7 and 10 after MI (Figure 5B). To determine whether silencing CRMP2 can change macrophage polarization, we evaluated the populations of CD11b^+^, CD86^+^, and CD206^+^ positive cells, representing total macrophages, M1 macrophages and M2 macrophages, respectively using immunohistochemistry. On day 3 after MI in the ApoE^−/−^ mice, CRMP2 silencing resulted in a marked decrease in M1 macrophages (Figure 5A and D) and a marked increase in M2 macrophages (Figure 5A and E) compared to the control siRNA treatment, whereas total macrophages did not differ significantly between the two treatments (Figure 5A and C). Similar changes were also observed on day 7 and 10 after MI (Figure 5C-E).

### CRMP2 silencing resulted in reduced fibrosis and accelerated resolution of inflammation in ApoE^−/−^ mice

**Figure 6:**
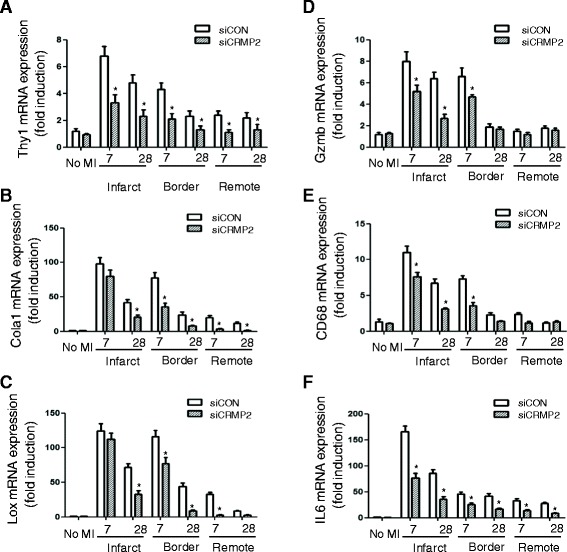
**CRMP2 RNAi reduced the expression of fibrosis and inflammation markers in ApoE** ^**−/−**^ **mice with MI.** Expression of different fibrosis and inflammation markers was analyzed by quantitative real-time PCR in myocardial samples from the infarct region (Infarct), infarct border zone (Border), and area remote to the infarct (Remote) 7 and 28 days after infarction. **A**. Fibroblast proliferation marker Thy1. **B**. Collagen 1a1 (Col1a1). **C**. Lysyl oxidase (Lox). **D**. Neutrophil marker granzyme B (Gzmb). **E**. Macrophage marker CD68. **F**. Proinflammatory cytokine interleukin-6 (IL-6). Results are expressed as fold induction ± SE over the uninjured values. *P < 0.05 compared to siCON group.

To determine whether silencing CRMP2 affects inflammation resolution and fibrosis, we analyzed different fibrosis and inflammation parameters by quantitative real-time PCR in the infarct region, border zone, and myocardial area remote to the infarct on day 7 and 28, respectively. The cardiac fibroblast proliferation marker Thy110 was strongly induced in the infarct region and border zone by 7 days after injury in siCON-treated mice, but was significantly lower in the siCRMP2 treated mice (Figure 6A). Both collagen 1α1 and lysyl oxidase were strongly induced in the infarct region and border zones in the siCON treated mice, particularly 7 days after infarction when the scar is being formed (Figure 6B and C). Silencing CRMP2 decreased the expression of these markers in the infarct region 28 days after surgery. In the border zone, a significant reduction could already be seen by 7 days. In the control siRNA treated animals, expression of fibrotic markers was also detected in the remote myocardial area, indicating the induction of interstitial fibrosis, although the expression was much lower than in the infarct region (Figure 6A-C). Animals treated with siCRMP2 showed lower levels of collagen 1α1 and lysyl oxidase in this area as well (Figure 6B and C).

We next analyzed the expression of different leukocyte population markers and inflammatory mediators. To determine the presence of neutrophils and macrophages, we measured the expression of granzyme B and CD68, respectively. We observed robust expression of both markers at 7 days after injury in the infarct region and border zone of both siCON- and siCRMP2-treated mice (Figure 6D and E). However, whereas expression of both granzyme B and CD68 was almost normalized in the border zone at 28 days in both groups, elevated expression persisted in the infarct region in the siCON-treated mice. Notably, levels of these markers were significantly lower in the infarct region of the siCRMP2-treated mice, suggesting a faster resolution of inflammation in these mice compared with the control group. Similarly, we observed a strong induction of the proinflammatory cytokine interleukin-6 in the infarct region and border zone of the siCON-treated mice 7 and 28 days after injury, but the IL-6 level was significantly lower in the siCRMP2-treated animals (Figure 6F). Collectively, these results indicate that silencing CRMP2 decreases fibrosis and accelerates the resolution of inflammation after myocardial infarction.

### Silencing CRMP2 reduced the extent of myocardial injury and decreased the mortality following MI in ApoE^−/−^ mice

**Figure 7:**
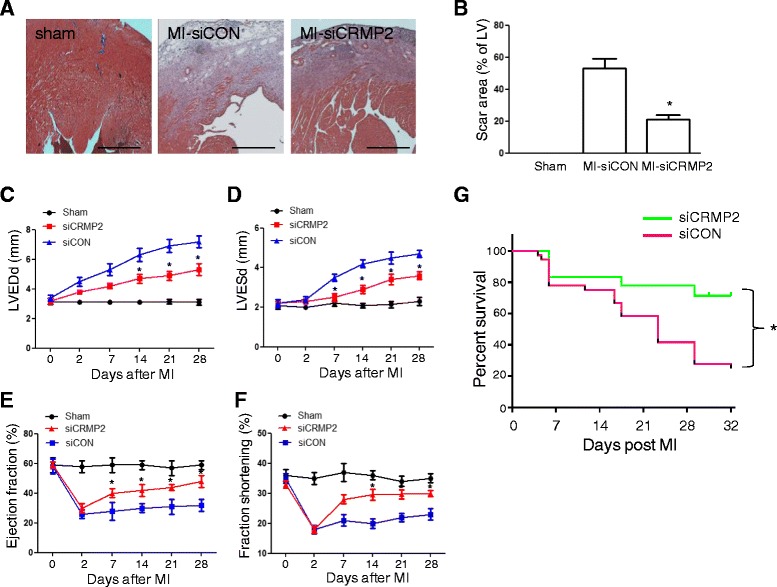
**Effects of CRMP2 silence on left ventricular fibrosis and heart function after MI in ApoE** ^**−/−**^ **mice. A**. Representative Masson’s trichrome staining revealed left ventricular fibrosis 4 weeks after MI (magnification: 4x). Scale bar indicates 500 μm. **B**. Quantitative analysis of the scar area (n = 10, *p < 0.05). **C**. Quantification of left ventricular end diastolic diameter (LVEDd). **D**. Quantification of left ventricular end systolic diameter (LVESd), **E**. Quantification of left ventricular ejection fraction. **F**. Quantification of fractional shortening (n = 30, *p < 0.05 vs. control). **G**. Kaplan–Meier survival curves in siCON and siCRMP2 groups with MI (n = 30 per group, *p < 0.05 vs. control).

To study the effects of CRMP2 knockdown in macrophages on the extent of fibrosis after MI, we performed Masson’s trichrome staining. As illustrated in Figure 7A, severe fibrosis was observed in the hearts of siCON group with MI, but treatment with siCRMP2 markedly reduced cardiac fibrosis. Quantitative measurement revealed that the scar area in the siCON group was 51.22 ± 3.93%. Conversely, the scar area in siCRMP2 group was 14.75 ± 3.12%, significantly less than that in the siCON group (P < 0.05) (Figure 7B).

Furthermore, echocardiogram studies were performed to evaluate the cardiac function after MI in both groups. Serial echocardiographic analysis indicating that siCRMP2 treatment manifested a trend towards improvement of cardiac performance after MI. Compared with sham group, MI increased the LVEDd and LVESd in both siCON and siCRMP2 groups, with significant less level in siCRMP2 group than that in siCON group (Figure 7C and D). Furthermore, the LVEF and FS were significantly enhanced in siCRMP2 group compared with siCON group (Figure 7E and F). These data suggested that siCRMP2 treatment decreased fibrosis and preserved cardiac function after MI. Moreover, the Kaplan–Meier survival curves indicated that siCRMP2 treatment significantly decreased the mortality after MI compared with the siCON treatment (Figure 7G).

## Discussion

With the recent insight into the molecular mechanisms governing macrophage heterogeneity, polarization and function [31], it has become feasible to modulate macrophage actions in interventions that might optimize healing of injured tissues. Identification of appropriate targets that modulate macrophages phenotypes is critical for this goal. In the present study, we provided the first evidence that CRMP2 is expressed in macrophages and its expression depends on the activated status of the cells. CRMP2 is predominantly expressed in M1 macrophages, and plays a role in macrophage polarization to M1 as silencing CRMP2 results in switch of macrophages from M1 to M2 not only *in vitro* but also in atherosclerotic mice with MI. Thus, modulation of CRMP2 expression may promote inflammation resolution and improve infarct healing.

Macrophages are key mediators of the immune response during inflammation. Plasticity and functional polarization are hallmarks of macrophages that result in the phenotypic diversity of macrophage populations [32]. Though many transcription factors, including PU.1, C/EBPβ, Runx1 and IRF8, are involved in lineage-specific transcriptional regulation during macrophage differentiation [33], only a small proportion of the macrophage transcriptome is altered by cell polarization [34]. Among the transcription factors, IRF5 is defined to determine commitment to the M1 macrophage lineage [23]. Another member of the IRF family, IRF4, known to inhibit IRF5 activation by competing for interaction with the adaptor Myd88, is reported to control the expression of prototypical mouse M2 macrophage markers [28]. We found that knockdown of CRMP2 reduced the expression of a number of M1 genes, including ccr7, cox2, tnf-α, cd86, il12b and cxcl10, and increased the expression of several M2 genes, including ym-1, arg-1, and il-10. These were associated with down-regulation of IRF5, without affecting the expression of IRF3 and IRF4. We further demonstrated that CRMP2-knockdown-induced M1 to M2 switch was reversed by overexpression of IRF5 in M1 macrophages. Therefore, it is postulated that CRMP2 may play a role in modulating macrophage polarization to M1 through, at least in part, regulating IRF5 expression. However, the underlying mechanism remains to be elucidated. It is postulated that CRMP2, which is localized in both cytoplasm and nucleus, may be directly involved in the transcriptional regulation of IRF5 or physically interact with IRF5 thereby stabilizing IRF5. These hypotheses need to be confirmed in future studies.

Enforcing the natural transition of M1 toward M2 macrophages in wounds may thus usher in resolution of inflammation and speed healing, especially if acute wound inflammation exists in the setting of an underlying chronic inflammatory disease. A prolongation of the inflammatory phase of wound healing inhibits regenerative processes and may compromise tissue integrity. Coronary ligation in ApoE^−/−^ mice allows the study of MI in the context of pre-existing chronic inflammation [29]. These atherosclerotic mice have impaired resolution of inflammation post-coronary ligation due to delayed M1 and M2 transition [30], thereby having a higher risk of developing heart failure post-MI, possibly due to compromised infarct healing [35]. We observed a significantly prolonged infiltration of M1 macrophages in the infarct of ApoE^−/−^ mice compared to that in wildtype mice. Intriguingly, the inflammatory macrophages in the infarct wound expressed CRMP2, which was localized at one pole outside of the nucleus.

To investigate the effect of CRMP2 knockdown on infarct healing in ApoE^−/−^ mice with MI, we used a lipidoid nanoparticle to deliver CRMP2 siRNA into the wound because employing the endocytic machinery of macrophages, intravenously administered nanoparticles are rapidly taken up by monocytes/macrophages, and accumulated in macrophages in atherosclerotic plaques [36,37], rendering inflammatory myeloid cells a prime target for *in vivo* RNAi [38-40]. In ApoE^−/−^ mice with MI, nanoparticle-mediated delivery of siRNAs has been shown to efficiently knockdown the target genes in macrophages in the infarct wound [18]. Consistently, we demonstrated that nanoparticle-mediated delivery of CRMP2 siRNA resulted in an efficient knockdown of CRMP2 in macrophages infiltrated in the wound. This was associated with a significantly decreased proportion of M1 but markedly increased proportion of M2 macrophages in the wound, whereas total macrophage percentage did not differ between the control and CRMP2 siRNA groups. We showed that in vivo knockdown of CRMP2 supported the resolution of inflammation, as numbers of inflammatory cells, including monocytes, neutrophils and macrophages were reduced. Moreover, CRMP2 RNAi decreased the extent of fibrosis, leading to an enhanced cardiac function recovery and decreased the mobility after MI.

In conclusion, we show that CRMP2 plays a role in macrophage polarization of M1 phenotype and CRMP2 RNAi resulted in a switch of M1 to M2 macrophages not only *in vitro* but also in ApoE^−/−^ mice with MI, leading to an promoted inflammation resolution, enhanced cardiac function recovery and decreased mobility after MI. Although our study bears some clinical relevance, the detailed physiologic and pathologic functions of CRMP2 have not been extensively characterized. Further studies defining the exact underlying mechanisms are needed.

## Abbreviations

CRMP2: Collapsin response mediator protein-2 (CRMP2)
qPCR: quantitative real-time PCR
RNAi: RNA interference
MI: Myocardial infarction
BMDMs: Bone marrow-derived macrophages
LVDs: Left ventricular systolic dimension
LVDd: Left ventricular diastolic dimension
AWT: Anterior wall thickness
PWT: Posterior wall thickness, LVEF, Left ventricular ejection fraction
FS: Fractional shortening
